# THE ROLE OF OUTDOOR LIGHT EXPOSURE ON SLEEP QUALITY IN OLDER INDIVIDUALS WITH FRAILTY OF DIFFERENT LIVING TYPES

**DOI:** 10.1093/geroni/igad104.3661

**Published:** 2023-12-21

**Authors:** Yi Chin Hsieh, Ching-Ju Chiu

**Affiliations:** National Cheng Kung University, Tainan, Tainan, Taiwan (Republic of China); National Cheng Kung University, Tainan, Tainan, Taiwan (Republic of China)

## Abstract

The study investigates the relationship between outdoor light exposure and sleep quality in older individuals with frailty in institutions and the community, where sleep disorders are common, and outdoor light exposure is inadequate.The study includes older individuals with frailty in community and institutions in Taiwan. Participants must be aged 65 or above and have a Clinical Frailty Scale score between 4 to 8. We collect sleep quality and outdoor light exposure data using questionnaires and actigraphy.The study includes 77 older individuals: 29 from day-care centers, 22 from conventional long-term care institutions, and 26 from unit care institutions. The average outdoor light exposure is 15 minutes per day, with an average Pittsburgh Sleep Quality Index (PSQI) score of 8, 74% sleep efficiency, and 125.8 minutes of wake after sleep onset. After controlling for multiple variables. Outdoor light exposure shows no relationship with PSQI, sleep efficiency, and wake after sleep onset.The average PSQI score is > 5, indicating poor subjective sleep quality. Additionally, Wake After Sleep Onset > 60 minutes, and sleep efficiency < 75%, suggesting poor objective sleep quality. The average outdoor light exposure time < 20 minutes, indicating a lack of outdoor light exposure. However, our study does not observe the effects of light exposure, possibly because the average daily light exposure is below the 30-minutes threshold. Older individuals with frailty experience poor sleep quality. It is possible that a longer duration of outdoor light exposure may be needed to observe its impact on sleep quality.

